# HSV-2 Infection as a Cause of Female/Male and Racial/Ethnic Disparities in HIV Infection

**DOI:** 10.1371/journal.pone.0066874

**Published:** 2013-06-18

**Authors:** Don C. Des Jarlais, Kamyar Arasteh, Courtney McKnight, David C. Perlman, Hannah L. F. Cooper, Holly Hagan

**Affiliations:** 1 Baron Edmond de Rothschild Chemical Dependency Institute, Beth Israel Medical Center, New York, New York, United States of America; 2 Department of Behavioral Sciences and Health Education, Emory University, Rollins School of Public Health, Atlanta, Georgia, United States of America; 3 College of Nursing, New York University, New York, New York, United States of America; University of Texas Health Science Center San Antonio Texas, United States of America

## Abstract

**Objectives:**

To examine the potential contribution of herpes simplex virus 2 (HSV-2) infection to female/male and racial/ethnic disparities in HIV among non-injecting heroin and cocaine drug users. HSV-2 infection increases susceptibility to HIV infection by a factor of two to three.

**Methods:**

Subjects were recruited from entrants to the Beth Israel drug detoxification program in New York City 2005-11. All subjects reported current use of heroin and/or cocaine and no lifetime injection drug use. A structured questionnaire was administered and serum samples collected for HIV and HSV-2 testing. Population-attributable risk percentages (PAR%s) were calculated for associations between HSV-2 infection and increased susceptibility to HIV.

**Results:**

1745 subjects were recruited from 2005-11. Overall HIV prevalence was 14%. Females had higher prevalence than males (22% vs. 12%) (p<0.001), African-Americans had the highest prevalence (15%), Hispanics an intermediate prevalence (12%), and Whites the lowest prevalence (3%) (p<.001). There were parallel variations in HSV-2 prevalence (females 86%, males 51%, African-Americans 66%, Hispanics 47%, Whites 36%), HSV-2 prevalence was strongly associated with HIV prevalence (OR  =  3.12 95% CI 2.24 to 4.32). PAR%s for HSV-2 as a cause of HIV ranged from 21% for Whites to 50% for females. Adjusting for the effect of increased susceptibility to HIV due to HSV-2 infection greatly reduced all disparities (adjusted prevalence  =  males 8%, females 11%; Whites 3%, African-Americans 10%, Hispanics 9%).

**Conclusions:**

Female/male and racial/ethnic variations in HSV-2 infection provide a biological mechanism that may generate female/male and racial/ethnic disparities in HIV infection among non-injecting heroin and cocaine users in New York City. HSV-2 infection should be assessed as a potential contributing factor to disparities in sexually transmitted HIV throughout the US.

## Introduction

There are substantial racial/ethnic disparities in HIV/AIDS in the United States, with African-Americans comprising 46% of newly diagnosed HIV infections despite comprising only 12% of the US population [1]. The Department of Health and Human Services, the White House Office of National AIDS Policy, and the National Institutes of Health have all prioritized reducing racial/ethnic disparities in HIV/AIDS in the US [2]–[4]. The racial/ethnic disparity is particularly striking for the “high-risk heterosexual transmission” category (in which the index case reports heterosexual activity with a person known to be HIV seropositive or to be at increased risk for HIV). In 2010, African-Americans comprised 67.4% of the 4416 heterosexual contact cases among males and 65.5% of the 8459 heterosexual contact cases among females.

There are also important female/male disparities among the heterosexual contact cases in the US. In 2010, there were a total of 12,875 newly reported heterosexual contact cases, of whom 4416 (34%) were among males and 8459 (66%) were among females. This disparity differs from that seen among female commercial sex work networks, in which a small number of females transmit HIV to large numbers of males, and differs from that seen in the “mature” heterosexual generalized epidemic in which HIV seropositive females outnumber HIV seropositive males, but typically only by a modest percentage, e.g., [5], [6].

Given the size of these disparities, it is likely that multiple causal factors are operating. The different causal factors: social structural factors such as male/female ratios, social network factors such as HIV prevalence among sexual networks, partnership factors such as the rate of sexual partner change, individual level factors such as the frequency of condom use, and biological factors such as the prevalence of sexually transmitted infections that may facilitate HIV transmission, may also be operating at different levels of analysis. Of note, individual level behaviors such as condom use vary little across racial/ethnic groups and typically do not explain racial/ethnic disparities [7], [8], [9]. One biological factor potentially contributing to the disparities that has not received much attention in the US is herpes simplex virus type 2 (HSV-2). HSV-2 increases susceptibility to HIV infection [10]–[12], and transmissibility of HIV from persons co-infected with both HSV-2 and HIV. [13], [14]. The likely causal mechanism for increased susceptibility to HIV infection is the recruiting of target cells for HIV infection in the genital mucosa [15]. The causal mechanism for increased transmissibility is believed to be increased HIV virus in genital secretions [16]. The relationship between HSV-2 and HIV infection has been extensively studied in sub-Saharan Africa. [10]–[12] The most recent evidence for HSV-2 associated with increased acquisition of HIV comes from the STEP vaccine trial with men-who-have-sex-with men, which found a three-fold increase in HIV acquisition among HSV-2 infected subjects compared to HSV-2 uninfected subjects [17].

In the US general population, HSV-2 seroprevalence among African-Americans is approximately three times as high (39.2%) as among White Americans (12.3%) and approximately twice as high among females (20.9%) compared to males (11.5%) [18]. These disparities in HSV-2 infection existed prior to the HIV/AIDS epidemic in the US [18]. Thus, the disparities in HSV-2 infection have the potential to generate female/male and racial/ethnic differences in sexually transmitted HIV infection. We report here on relationships between HSV-2, HIV, sex and race/ethnicity, among non-injecting heroin and cocaine users in New York City. Non-injecting cocaine users may be considered to be at particularly high risk for heterosexual acquisition of HIV [19]. Specifically, we utilize population attributable risk percentages to assess HSV-2 as a factor that may generate female/male and racial/ethnic disparities in HIV infection among these non-injecting drug users.

## Methods

The data reported here are derived from ongoing analyses of data collected from drug users entering the Beth Israel Medical Center drug detoxification program in New York City. The methods for this “Risk Factors” study have been previously described in detail [20], [21], so only a summary will be presented here. The Beth Israel detoxification program serves New York City, with approximately half of its patients living in Manhattan, one quarter in Brooklyn, one-fifth in the Bronx, and the remainder (i.e., 5%) living elsewhere. Patients enter the program voluntarily. There have been no changes in the requirements for entrance into the program over the time periods for the data presented here.

Both injecting and non-injecting drug users entering the detoxification program are eligible to participate in the study. The present analyses include only persons who reported that they have never injected illicit drugs (never-injecting drug users or NIDUs). As we were interested in the relationship of HSV-2 infection to sexual transmission of HIV, including persons who might have become infected with HIV through drug injecting transmission would have introduced considerable measurement error into the analyses. Hospital records and the questionnaire results are checked for consistency on route of drug administration and subjects are examined for physical evidence of injecting. The data presented here are from subjects who participated in the study from 2005 to 2011.

Research staff visited the general admission wards of the program in a preset order and examined all intake records of a specific ward to construct lists of patients admitted within the prior three days. All of the patients on the list for the specific ward were then asked to participate in the study. Among patients approached by our interviewers, willingness to participate has been more than 95%. After all the patients admitted to a specific ward in the three-day period had been asked to participate and interviews have been conducted among those who agreed to participate, the interviewers moved to the next ward in the preset order. Because there was no relationship between the assignment of patients to wards and the order that the staff rotated through the wards, these procedures should produce an unbiased sample of persons entering the detoxification program.

A structured questionnaire covering socio-demographic characteristics, drug use, sexual risk behavior, and use of HIV prevention services was administered by a trained interviewer. Race/ethnicity was assessed by asking participants with which racial/ethnic group they identified. African-American, Hispanic and White were the most common groups given. A small number (<0.05%) of participants gave other responses, but they were not included in the analyses presented here. Most HIV risk behavior questions referred to the six months prior to the interview.

After completing the interview, the participant was seen by an HIV counselor for pretest counseling and serum collection. HIV testing was conducted at the New York City Department of Health Laboratory using a commercial, enzyme-linked, immunosorbent assays (EIA) test with Western blot confirmation (BioRad Genetic Systems HIV-1-2+0 EIA and HIV-1 Western Blot, BioRad Laboratories, Hercules, CA). HSV-2 testing was conducted for all subjects beginning in 2005 and was performed by BioReference Laboratories using the Focus HerpeSelect 1 and 2 ELISA. We used an index value of 1.1 or greater for classifying a subject as HSV-2-seropostive.

Participants were included in this analysis if they reported that they had never injected illicit drugs. As we were primarily interested in the relationship of HSV-2 infection to heterosexual transmission of HIV, subjects who reported male with male sexual behavior without also reporting male with female sexual behavior were excluded from the analyses. We did include subjects who reported both male with male and male with female sexual behavior, as these subjects may well be contributing to heterosexual transmission. As we were interested in racial/ethnic disparities in HIV infection, we limited the analyses to subjects who reported their race/ethnicity as White, African-American and Hispanic. The numbers of subjects who reported other specific ethnicities were too small for statistical testing. Subjects were permitted to participate in the study multiple times, though only once per year. For these analyses, if subjects had multiple interviews during 2005-2011, we used the first interview. The design of the study is thus a cross-sectional survey.

We calculated “population attributable risk percentages” (PAR%s)[22] for HSV-2 as a causal factor for increased susceptibility to HIV infection in the 2005-2011 sample. HSV-2 biologically increases susceptibility to HIV infection by a relative risk of 2 – 3 [10], [23], or higher [12], [24]. We used a Relative Risk of 2.5 and the formula (Relative Risk – 1)/(Relative Risk) × (Prevalence of the risk factor in the study sample) [25] to calculate the PAR% for increased susceptibility to HIV due to the presence of HSV-2 infection.

The Stata statistical programs (StataCorp, College Station, TX) were used for statistical analyses.

The study was approved by the Beth Israel Medical Center Institutional Review Board.

## Results

### Subject sociodemographic characteristics and drug and sexual risk behaviors

Table 1 presents the demographic characteristics, drug use and sexual risk behaviors (six months prior to interview), and HIV serostatus for the subjects. Significant female/male and racial/ethnic disparities in HIV prevalence existed, with females having higher HIV prevalence than males (22% vs 12%), and a pattern of lowest HIV prevalence among Whites (3%), intermediate prevalence among Hispanics (12%), and highest prevalence among African-Americans (15%). Note that HIV seropositive subjects tended to report less sexual risk behavior than HIV seronegative subjects. Ninety-nine percent of these subjects had previously been tested for HIV and presumably the HIV seropositives reduced their risk behavior in order to reduce the likelihood that they would transmit HIV to sexual partners [26]. This “reverse causation” precludes us from assessing whether sex or racial/ethnic differences in current risk behavior might explain the sex and racial/ethnic differences in HIV seroprevalence. It would be necessary to have risk behavior data for time just prior to seroconversion to address the question of whether differences in risk behavior explained the disparities in HIV prevalence by sex and race/ethnicity.

**Table 1:** Demographic characteristics, drug use and sexual risk behaviors among never-injecting drug users, New York City, 2005–2011.

	Total n (%)	HIV- n(%)	HIV+ n(%)
Total	1745 (100)	1501 (86)	244 (14)
Sex*			
Male	1322 (76)	1169 (88)	153 (12)
Female	423 (24)	332 (78)	91 (22)
Race/Ethnicity*			
White	92 (5)	89 (97)	3 (3)
African-American	1207 (69)	1020 (85)	187 (15)
Hispanic	446 (26)	392 (88)	54 (12)
Age			
<35	216 (13)	192 (89)	24 (11)
35 or older	1496 (87)	1283 (86)	213 (14)
Multiple sex partners *			
No	1104 (63)	934 (85)	170 (15)
Yes	641 (37)	567 (88)	74 (12)
Unsafe sex w/primary partner*			
No	965 (56)	793 (82)	172 (18)
Yes	770 ( 44)	702 (91)	68 (9)
Unsafe sex w/ casual partner*			
No	1416 (82)	1204 (85)	212 (15)
Yes	307 (18)	281 (92)	26 (8)
Drug use past 6 months			
Crack cocaine*	1266 (73)	1055 (83)	211 (17)
Cocaine*	708 (41)	639 (90)	69 (10)
Heroin*	670 (38)	609 (91)	61 (9)

Note: Percentages in the first column are expressed relevant to total subjects,

whereas percentages in the second and third columns are expressed relevant to the row total (number of subjects in the specific subgroup).

* significant difference (p<0.05) in HIV seropositivity by chi-squared test.

Table 2 shows HIV prevalence, HSV-2 prevalence and the odds ratios for the associations between HSV-2 and HIV for all subjects, males and females, and by race/ethnicity. The patterns in HIV and HSV-2 prevalence are parallel, and the odds ratios are all significant. Females tended to have the highest odds ratio, though there was overlap in all of the 95% confidence intervals.

**Table 2:** HIV and HSV-2 prevalence and associations by female/male and race/ethnicity among never-injecting drug users, New York City, 2005-2011.

	HIV+n (%)	HSV-2+n (%)	OR (95% CI)
Total	244 (14)	1037 (59)	
Sex			
Male	153 (12)	672 (51)	2.40 (1.67–3.45)
Female	91 (22)	365 (86)	9.0 (2.16–37.75)
Race/Ethnicity			
White	3 (3)	33 (36)	3.74 (0.33–42.92)
African-American	187 (15)	793 (66)	2.78 (1.87–4.14)
Hispanic	54 (12)	211 (47)	3.33 (1.77–6.23)

Table 3 shows the observed HIV prevalence, population attributable risk percentages, and adjusted HIV prevalence among the subjects. The population attributable risk percentages are based on HSV-2 as a biological cause of increased susceptibility for acquisition of HIV, with a relative risk of 2.5. The adjusted prevalences are the calculated prevalence rates that would have been observed if HSV-2 did not increase susceptibility to HIV in this population, using the formula (adjusted prevalence  =  observed prevalence times [1 – PAR %]). Adjusting for the increased susceptibility effect of HSV-2 on acquisition of HIV greatly reduces the female/male disparity from a difference of 10% to a difference of 3%. It also essentially removes the African-American/Hispanic disparity, and greatly reduces the African-American/White and Hispanic versus White disparities in HIV prevalence. The difference between African-Americans and Whites is reduced from 12% to 7% and the difference between Hispanics and Whites is reduced from 9% to 6%. Overall, adjusting for the biological effect of HSV-2 on susceptibility to HIV infection reduced the different disparities by a third (Hispanics vs. Whites), by approximately half (African-Americans vs. Whites), and by over two thirds (females vs. males).

**Table 3:** Observed HIV prevalence, population attributable risk percentages, and adjusted HIV prevalence among never-injecting drug users, New York City, 2005–2011.

	Observed prevalence%	PAR%	*Adjusted prevalence%
Sex			
Male	12	29	8
Female	22	50	11
Race/Ethnicity			
White	3	21	3
African-American	15	38	10
Hispanic	12	26	9

* Adjustment removes the effect of increased susceptibility to HIV infection.

due to HSV-2 infection. Using formula: Adjusted prevalence  =  Observed.

prevalence (1 – PAR%).

## Discussion

We observed significant sex and racial/ethnic disparities in HSV-2 infection and in HIV infection among non-injecting heroin and cocaine users in New York City. Adjusting for the biological effect of HSV-2 increasing susceptibility to HIV infection greatly reduced the female/male disparity and the disparities between Whites versus African-Americans and Hispanics. The disparities in HSV-2 infection preceded the HIV/AIDS epidemic in the US, so that the biological mechanism of increased susceptibility can thus explain much of the disparities in HIV infection [27]. As noted in the Methods section, the design of this research is a cross-sectional survey. Caution must be exercised in drawing causal inferences from cross-sectional data, but there are several factors supporting sex and racial/ethnic disparities in HSV-2 as a cause of sex and racial/ethnic disparities in HIV infection. First, sex and racial/ethnic disparities in HSV-2 infection existed in the US population in the 1970s [28], well before large-scale introduction of HIV into the heterosexual population. So the temporal order of HSV-2 disparities preceding HIV disparities is correct for HSV-2 disparities as a cause of HIV disparities. Second, that HSV-2 infection increases susceptibility to HIV infection has been well established through longitudinal studies [10]. Thus, while our cross-sectional data cannot prove a causal relationship between disparities in HSV-2 and disparities in HIV, we believe that, in conjunction with previous research on HSV-2 as a cause of increased susceptibility to HIV, our data illustrate a causal relationship.

It is likely that there are multiple causes of the female/male and racial/ethnic disparities in HIV/AIDS. HSV-2 is not a complete explanation for these disparities, but the data presented here for heterosexual non-injecting drug users suggest that HSV-2 should be considered when examining female/male and race/ethnic disparities in sexual transmission of HIV in the US.

Limitations. Several limitations of the present study should be considered. First, the data presented here are from subjects recruited at a single site. However, the data are consistent with HIV prevalence among non-injecting drug users recruited from community settings in New York in 2004 [29] and with the HIV prevalence among “high risk heterosexuals” in the 2006-07 National HIV Behavioral Survey conducted in New York City. That study recruited subjects from high-risk neighborhoods and found an overall HIV prevalence of 8.6% [30].

The data presented here are also consistent with the recent study showing unexpectedly high HIV incidence among women in six US cities [31] including New York. Finally, the prevalence of HSV-2 nationally among African-Americans is approximately twice that among Whites in the US general population [18], so that our findings may also help explain racial/ethnic disparities in sexual transmission of HIV in other parts of the country, though this hypothesis needs to be tested in additional research.

Second, the research design used in this study was a cross sectional survey, so it was not possible to observe new HSV-2 and new HIV infections in individual subjects or identify the specific risk behaviors associated with seroconversion. Data on risk behavior at the time of HIV seroconversion would permit examining whether racial/ethnic differences in risk behaviors contribute to the racial/ethnic disparities in HIV prevalence which we were not able to do in this study. Seroconversions and associated risk behaviors could have been observed in a cohort study however an extremely large study would be needed. We estimate that incidence among these NIDUs was approximately 1-2/100 person-years overall, so that a very large number of subjects would have to be followed over a long time period to detect significant racial/ethnic differences in incident HIV infections.

Third, the effect of HSV-2 on increased susceptibility to HIV infection is undoubtedly an important contributing factor to the high HIV prevalence (14% overall) we observed in these NIDUs, but clearly is not a complete explanation for this relatively high prevalence. Other factors, including a biological effect of HSV-2 on the transmission of HIV and transmission from HIV seropositive drug injectors to non-injecting drug users are likely to be operating. Unsafe sexual behavior would also be a necessary causal factor for acquisition of HIV among our subjects.

These limitations are important, but it is difficult to envision how they would have created the sex and racial/ethnic patterns in HSV-2 and HIV serostatus that we observed. Rather it is much more likely that these patterns are sufficiently strong that we observed them despite the limitations of the study.

## Conclusions

Adjusting for a causal pathway of HSV-2 increasing susceptibility to HIV essentially eliminates the female/male disparities and greatly reduces the racial/ethnic disparities in HIV infection among these New York non-injecting heroin and cocaine users. The higher rates of HSV-2 among racial/ethnic minorities have been observed since the late 1970s in the US [27]. Racial/ethnic disparities in HSV-2 would have preceded large-scale introduction of HIV into the population of heterosexual non-injecting heroin and cocaine users in New York City. Thus, the racial/ethnic disparities in HIV infection among these non-injecting drug users would have developed in part from prior racial/ethnic disparities in HSV-2 infection. While disparities in HSV-2 prevalence would be a proximal cause of the disparities in HIV infection, the factors that generated the original disparities in HSV-2 would then also need to be considered as distal causes of the disparities in HIV infection.

Finally, there is a clear need to implement interventions to reduce HSV-2 related HIV transmission among non-injecting drug users and their sexual partners. There are therapies that suppress HSV-2 infection and reduce the frequency of outbreaks [32]. Unfortunately, suppressive therapy for HSV-2 does not reduce the transmission of HIV [33]. It is likely that rather than any single intervention, “combined” prevention programming would be more effective in reducing HSV-2 related transmission of HIV. Combined programming might include condom social marketing programs, psychosocial programs to reduce sexual risk behavior among drug users [34], provision of drug dependence treatment, pre-exposure prophylaxis for HSV-2 seropositive/HIV seronegative persons and “treatment as prevention” [35] for HIV seropositive sexual partners of HSV-2 seropositive/HIV seronegative persons.

Locating and working with HSV-2 seropositive drug users at risk for HIV and locating and working with their possibly HIV seropositive partners, would require substantial resources, but given the increased susceptibility to HIV associated with HSV-2 infection, and its relationship to racial/ethnic disparities in HIV infection, the additional costs would be fully justified.
